# Genome-Wide Identification, Evolutionary and Expression Analyses of the *GALACTINOL SYNTHASE* Gene Family in Rapeseed and Tobacco

**DOI:** 10.3390/ijms18122768

**Published:** 2017-12-20

**Authors:** Yonghai Fan, Mengna Yu, Miao Liu, Rui Zhang, Wei Sun, Mingchao Qian, Huichun Duan, Wei Chang, Jinqi Ma, Cunmin Qu, Kai Zhang, Bo Lei, Kun Lu

**Affiliations:** 1 College of Agronomy and Biotechnology, Southwest University, Chongqing 400715, China; fyh1212@email.swu.edu.cn (Y.F.); yumengna1024@163.com (M.Y.); monky1117@email.swu.edu.cn (M.L.); z2247319@email.swu.edu.cn (R.Z.); reginasw@163.com (W.S.); absorbessence@163.com (M.Q.); dhcspring@163.com (H.D.); cw12345678@email.swu.edu.cn (W.C.); mjq2014@email.swu.edu.cn (J.M.); lion4302@163.com (C.Q.); 2 Academy of Agricultural Sciences, Southwest University, Chongqing 400715, China; 3 Key Laboratory of Molecular Genetics, China National Tobacco Corporation, Guizhou Academy of Tobacco Science, Guiyang 550081, China; 4 Upland Flue-Cured Tobacco Quality and Ecology Key Laboratory of China Tobacco, Guizhou Academy of Tobacco Science, Guiyang 550081, China

**Keywords:** galactinol synthase, *Brassica napus*, *Nicotiana tabacum*, evolution, expression

## Abstract

Galactinol synthase (GolS) is a key enzyme in raffinose family oligosaccharide (RFO) biosynthesis. The finding that GolS accumulates in plants exposed to abiotic stresses indicates RFOs function in environmental adaptation. However, the evolutionary relationships and biological functions of GolS family in rapeseed (*Brassica napus*) and tobacco (*Nicotiana tabacum*) remain unclear. In this study, we identified 20 *BnGolS* and 9 *NtGolS* genes. Subcellular localization predictions showed that most of the proteins are localized to the cytoplasm. Phylogenetic analysis identified a lost event of an ancient GolS copy in the Solanaceae and an ancient duplication event leading to evolution of *GolS4/7* in the Brassicaceae. The three-dimensional structures of two GolS proteins were conserved, with an important DxD motif for binding to UDP-galactose (uridine diphosphate-galactose) and inositol. Expression profile analysis indicated that *BnGolS* and *NtGolS* genes were expressed in most tissues and highly expressed in one or two specific tissues. Hormone treatments strongly induced the expression of most *BnGolS* genes and homologous genes in the same subfamilies exhibited divergent-induced expression. Our study provides a comprehensive evolutionary analysis of *GolS* genes among the Brassicaceae and Solanaceae as well as an insight into the biological function of *GolS* genes in hormone response in plants.

## 1. Introduction

Abiotic stress, such as drought, salinity, low temperature, can seriously affect the normal growth and development of plants, especially the yield of crops [1,2]. In response to environmental changes, plants formed a set of physiological, biochemical, cellular and molecular mechanisms to deal and cope metabolically with water deficit periods [3]. To improve the stress resistances and reduce the possibly damages from abiotic stresses, plants have evolved complex strategies, such as the accumulation of certain small soluble molecules (such as trehalose, proline, tryptophan, polyamines and glycyl betaine), to increase the osmotic pressure in cells [4,5]. Acting as osmoprotectants, raffinose family oligosaccharides (RFOs), including raffinose, stachyose and verbascose, play key roles in plant growth and development. Additionally, the accumulation of RFOs is associated with stressful environmental conditions, especially desiccation tolerance [6,7,8,9,10]. In addition, RFOs also act as signaling molecules during pathogen attack and wounding and are used in carbohydrate catabolism to generate energy during germination [11,12,13,14,15].

Galactinol synthase (GolS; EC: 2.4.1.123), the key enzyme in the production of RFOs, is a small representative class of the eukaryotic glycosyltransferase family of enzymes (GTs; EC: 2.4.x.y) and involved in the biosynthesis of diverse sugars. As a key regulator of carbon-partitioning, it also catalyzes the first step in the RFO biosynthesis, i.e., from UDP-galactose (uridine diphosphate-galactose) and inositol to galactinol (α-d-galactosyl-1-l-myo-inositol) [13,16]. Hence, GolS is regarded as an important regulator of RFOs that imparts the osmoprotectant function to RFOs [4]. Previous researches revealed the positive relationship between GolS activity and plant abiotic stress tolerance. Expression of *OsGolS* increase in response to cold treatments and osmotic stress in rice (*Oryza sativa*) seedlings [17]. In *Arabidopsis thaliana*, *AtGolS1*, *AtGolS2* and *AtGolS3* were expressed in mature seeds and are also highly induced by abiotic stresses [4]. *AtGolS1* and *AtGolS2* are induced by drought and salt stress but not cold stress, while *AtGolS3* is only induced by cold stress. In maize (*Zea mays*), *ZmGolS1* has low expression in most tissues, while *ZmGolS2* accumulates in germinating seeds subjected to dehydration stress and *ZmGolS3* predominated in seeds prior to maturation desiccation [18]. In addition, galactinol and raffinose accumulation are related to plant cell protection against oxidative damage caused by many types of stressors and are positively correlated with the biosynthesis of abscisic acid (ABA) [19,20]. These studies all suggested that GolSs play crucial roles in plant abiotic stresses and hormone response.

So far, *GolS* genes have been identified and studied in several plant species at genome-wide level and many of *GolS* genes are up-regulated during abiotic and hormone treatments [4,12,14,15,16,17,18,19,20,21,22,23]. Previous evolutionary research suggested that *GolS* might have evolved from an ancestral fungal sequence (this gene has been lost in the process of evolution) and the role of *GolS* has being largely seed–plant–specific, the enzyme has been retained and evolved among angiosperms while not retained or differently evolved among other taxa [24]. In the phylogenetic tree of GolS proteins in flowering plants, the GolS in gymnosperm *Pseudotsuga menziesii* is the base and separation between monocot and dicot clades is distinct, suggesting the two clades might originated from a common ancestor. Though previous study observed that all GolS in monocots are clustered in a narrow clade whereas the diversification in dicots is greater [24], the evolutionary relationships of GolS proteins and their biological meanings in dicots, especially allopolyploid dicots remain unclear.

Rapeseed (*Brassica napus*) is one of the most important allopolyploid crops worldwide. It is formed by two progenitor species, *Brassica rapa* and *Brassica oleracea* [25,26]. Tobacco (*Nicotiana tabacum*) is an important allopolyploid plant for evolution and gene function characterization [27,28]. To unravel the evolutionary relationships of GolSs in allopolyploid dicots, we used the rapeseed and tobacco as model plants in this study and identified GolS family members in three Brassicaceae species (*B. napus*, *B. rapa* and *B. oleracea*) and three Solanaceae species (*N. tabacum*, *Solanum lycopersicum* and *Solanum tuberosum*). Then, we investigated the evolutionary relationships among GolS members in Brassicaceae and Solanaceae species and evaluated the predicted three-dimensional structures of representative GolS members from *B. napus* and *N. tabacum*. In addition, we determined the spatio-temporal and hormone response expression patterns of these *GolS* genes. Our study provides a comprehensive evolutionary analysis of *GolS* genes among the Brassicaceae and Solanaceae and a basis for subsequent studies examining the role of GolS in regulating plant tolerance.

## 2. Results

### 2.1. Identification of GolS Genes in Rapeseed and Tobacco

Using seven AtGolS proteins from *A. thaliana* as the query sequences, we identified 20 *BnGolS* genes and nine *NtGolS* genes (Table 1). All of the identified GolS proteins share a common Pfam (PF01501.18), which is a conserved domain of the glycosyl transferase 8 family. The nomenclature for *BnGolS* (*BnGolS1-1* to *BnGolS7-2*) and *NtGolS* (*NtGolS1-1* to *NtGolS 2-6*) genes was based on the corresponding *AtGolS* orthologues (Table 1). Each *AtGolS* corresponds to multiple *GolS* homologs in *B. napus* except *AtGolS5*, no *B. napus* homologs was identified, while only *AtGolS1* and *AtGolS2* orthologues were identified in *N. tabacum* (Table 1). The number of amino acid residues in the BnGolS proteins ranged from 121 (BnGolS7-1) to 342 (BnGolS1-1 and BnGolS1-4) and the relative molecular weights (MWs) ranged from 13.74 kDa (BnGolS7-1) to 39.26 kDa (BnGolS1-1). Theoretical pI (isoelectric point) values ranged from 4.69 (BnGolS4-1) to 7.03 (BnGolS3-2) and only that of BnGolS3*-2* was higher than 7 in *B. napus*. The NtGolS proteins possessed 223 (NtGolS1-3) to 343 (NtGolS 1-1 and NtGolS1-2) amino acid residues, with relative molecular weights ranging from 25.26 kDa (NtGolS1-3) to 41.40 kDa (NtGolS2-2). Theoretical pI values ranged from 5.18 (NtGolS2-3) to 7.03 (NtGolS2-6) and only NtGolS2-6 exhibited a pI of >7. No transmembrane transport and signal peptide sequences were identified in the GolS proteins from rapeseed or tobacco (Table 1). Subcellular localization predictions for BnGolS and NtGolS suggest that only BnGolS3-2 localizes to mitochondria, while the other 28 GolS proteins all localize to the cytoplasm (Table 1).

### 2.2. Phylogenetic Analysis of BnGolS and NtGolS Proteins

**Figure 1:**
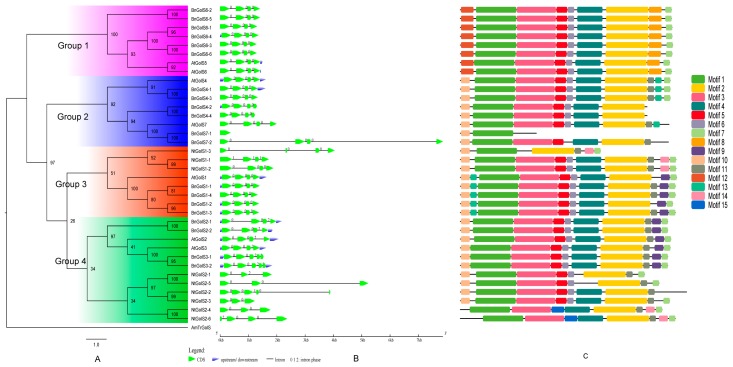
Phylogenetic, gene structure and conserved motifs of GolS proteins in *A. thaliana*, *B. napus* and *N. tabacum*. (**A**) Amino acid sequences of AtGolS, BnGolS and NtGolS were aligned using MUSCLE. The phylogenetic tree was constructed with the online PhyML server with bootstrap analysis (100 replicates) and displayed using FigTree v1.4.0. The 36 GolS proteins from *A. thaliana*, *B. napus* and *N. tabacum* (GolS in *A. trichopoda* as an outgroup) clustered into four distinct groups; (**B**) Gene structures were generated by the Gene Structure Display Server. Exons (CDS) and introns are shown with green wedges and black lines, respectively. Numbers 0, 1 and 2 represent the introns in phases 0, 1 and 2, respectively. The scale bar represents 1.0 kb. At: *A. thaliana*; Bn: *B. napus*; Nt: *N. tabacum*; AmTr: *A. trichopoda*. (**C**) Conserved motifs in AtGolS, BnGolS and NtGolS proteins were identified by MEME. A colored box indicates the different motifs that are numbered along the bottom.

To reveal the evolutionary relationships among *GolS* gene family members in *B. napus*, *N. tabacum* and *A. thaliana*, we performed multiple sequence alignment to 29 GolS protein sequences, using AmTrGolS (GolS in *Amborella trichopoda*) as an outgroup. Based on the lowest values of Akaike Information Criterion (AIC) and Bayesian Information Criterion (BIC) in Modelgenerator (Table S1), the was selected for construction of the maximum likelihood (ML, 100 bootstrap replicates) and Bayesian (BI) trees. According to previous phylogenetic analysis [29], GolS proteins were divided into four groups, including the GolS5 and GolS6 clade (Group 1), the GolS4 and GolS7 clade (Group 2), the GolS1 clade (Group 3) and GolS2 and GolS3 clade (Group 4) (Figure 1A). Group 1 and Group 2 contained 12 BnGolSs, while Group 3 and Group 4 possessed 8 BnGolSs and all NtGolSs. In Group 2, the evolutionary relationship between BnGolS and AtGolS was closer than that between NtGolS and AtGolS, which is consistent with the evolutionary relationship of these species. Since *Arabidopsis* and *B. napus* both belong to Brassicaceae, Rosidae and their split time might be 13 million years ago (MYA) [30]. Tobacco belongs to Solanaceae, Asterids, it’s ancestor had been diverged from ancestor of Rosidae about 83–123 MYA [31]. Phylogenetic analyses of 83 protein-coding and rRNA genes from the plastid genome for 86 species of seed plants [32] also proven the closer relationship between *Arabidopsis* and *B. napus*. All BnGolS and NtGolS proteins except for BnGolS7-1 possessed DxD and HxxGxxKPW motifs, the conserved motifs of GT members [33,34]. Both of the BnGolS6 and NtGolS2 subfamilies have six members, suggesting a notable expansion of these gene subfamilies compared with the other BnGolS and NtGolS subfamilies. Gene loss was also observed for GolS5 in *B. napus* and GolS3/4/5/6/7 in *N. tabacum*, since no orthologues of these proteins were found in the aforementioned species.

**Figure 2:**
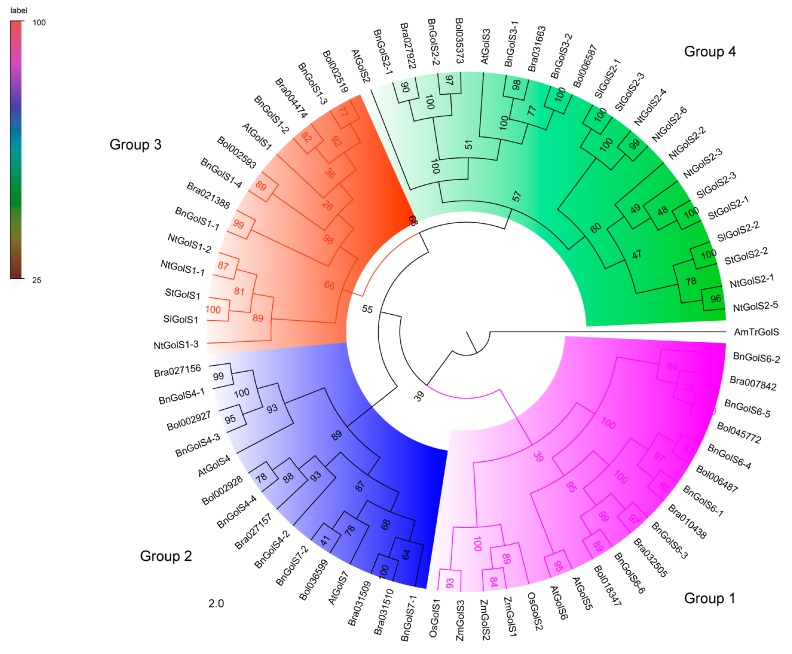
Phylogenetic relationship among GolS proteins in plants. The ML tree was generated with bootstrap analysis (100 replicates) using aligned GolS protein sequences from *A. thaliana*, *B. napus*, *B. rapa*, *B. oleracea*, *N. tabacum*, *S. lycopersicum*, *S. tuberosum*, *O. sativa* and *Z. mays* (GolS in *A. trichopoda* as an outgroup) using the online PhyML server. The tree was displayed with FigTree v1.4.0. GolS proteins in the phylogenetic tree clustered into four groups (Group 1, Group 2, Group 3 and Group 4). At: *A. thaliana*; Bn: *B. napus*; Bra: *B. rapa*; Bol: *B. oleracea*; Nt: *N. tabacum*; Sl: *S. lycopersicum;* St: *S. tuberosum*; Os: *O. sativa*; Zm: *Z. mays*; AmTr: *A. trichopoda.*

**Figure 3:**
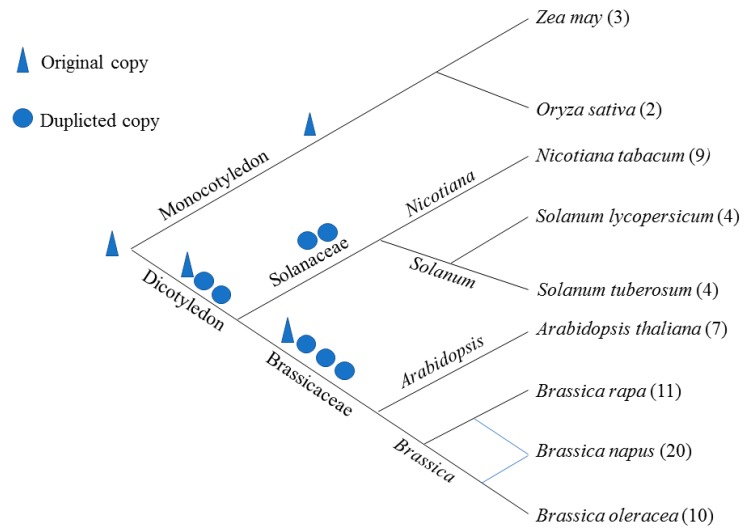
Inferred origin and evolutionary relationship of *GolS* genes and their copy number change among nine plants. The digits represent the number of *GolS* genes in plants species. Triangle represents the original genes of *GolS* in plants, while the circle represents the duplicated genes. The blue line indicates that *B. napus* is formed by *B. rape* and *B. oleracea*.

To further investigate the evolutionary relationships among GolS proteins in Brassicaceae and Solanaceae, a larger ML tree was constructed based on 70 GolS proteins from *A. thaliana*, *B. napus*, *B. rapa*, *B. oleracea*, *N. tabacum*, *S. lycopersicum*, *S. tuberosum*, *O. sativa* and *Z. mays* (AmTrGolS as an outgroup) (Figure 2). Considering that GolS formed a unique clade within the GT family [35,36], we also divided the 70 GolS members into four groups based on previous study [29] and gene copy number comparison (Figure 3). Group 4 (GolS2 and GolS3 clade) was the largest clade, containing 22 GolS members, including 10 GolSs from Brassicaceae and 12 GolSs from Solanaceae species, while group 3 was the smaller clade (GolS1), including 9 GolSs from Brassicaceae and 5 GolSs from Solanaceae species. Group 2 was the Brassicaceae-specific group, which contained 15 GolSs (GolS4 and GolS7 clade), the 15 GolSs only found in the Brassicaceae. Group 1 (GolS5 and GolS6 clade) contained 14 GolSs from Brassicaceae, 5 GolSs from monocots and no GolS from Solanaceae. We were unable to identify orthologues of AtGolS5 in the three *Brassica* species and orthologues of the AtGolS3/4/5/6/7 in the three Solanaceae species, indicating that gene loss of GolS is more pronounced in Solanaceae than in *Brassica* species. Each of the AtGolSs and its orthologues from the three *Brassica* species consistently formed a Brassicaceae cluster first, suggesting that GolS members in *B. napus*, *B. oleracea* and *B. rapa*, are highly homologous. Similarly, close evolutionary relationships were also found among the GolS families from *N. tabacum*, *S. lycopersicum* and *S. tuberosum*. Orthologues of AtGolS further clustered into a larger clade (Figure 2). For example, BnGolS1-1 and Bra021388 and BnGolS1-4 and Bol002593 formed two small *Brassica* clusters in terms of evolutionary time, then formed the larger *Brassica* GolS1 cluster with BnGolS1-2, Bra004474, BnGolS1-3 and Bol002519 and then the Brassicaceae GolS1 cluster formed with AtGolS1. Finally, the large GolS1 clade was formed by grouping the Brassicaceae and Solanaceae GolS1 branches. The relationship among GolS genes is in accordance with the evolutionary relationship among the Brassicaceae and Solanaceae families. In addition, the group 1 GolS members from monocots clustered in a clade with GolS5/6 members from Brassicaceae species, which are dicots. This clade represents the most ancient GolS members, which have been lost in the Solanaceae. Members of Brassicaceae in this clade (AtGolS5/6) clustered together and no orthologues of AtGolS5 were found in the three *Brassica* species. The GolS2/3 members from Brassicaceae clustered together to form a large clade with the GolS2 members from Solanaceae, suggesting that members of GolS3 may be either newly produced genes in Brassicaceae or may have been lost in Solanaceae (Figure 3). In Group 2, GolS4/7 are the most likely newly evolved Brassicaceae-specific genes, since GolS members in this clade were only identified in *A. thaliana* and three *Brassica* species.

### 2.3. Gene Structure and Conserved Motif Analyses

To confirm the exon/intron structures of *AtGolS*, *BnGolS* and *NtGolS* genes, we aligned the cDNAs with their corresponding genomic sequences. The gene structures among *BnGolS* genes are highly conserved and have high similarity with *AtGolSs*. However, the structures of *NtGolS* genes were noticeably different from each other and from the homologous *AtGolS* genes (Figure 1B). Interestingly, most of the *NtGolS* genes have one or two introns that are longer than their exons but the reverse trend was found in most of the *BnGolS* genes, including *BnGolS2-1*, *BnGolS2-2* and *BnGolS7-2*. All of the *AtGolSs* genes and most of the *BnGolS* genes are composed of four exons and three introns, with a few exceptions: *BnGolS7-1* only has one exon and no introns and *BnGolS3-1* and *BnGolS3-2* each have five exons and four introns (Figure 1B). The exons and introns of duplicated *NtGolS* genes varied substantially.

To better understand the structural diversity of BnGolS and NtGolS proteins, online MEME/MAST tools were used to analyze the conserved motifs [37]. Fifteen putative protein motifs were predicted using the MEME program and uploaded into MAST for motif detection. We found that motif 1 was observed in all 36 GolS proteins, motif 2 was observed in all GolS proteins except BnGolS7-1 and motif 3 was observed in most GolS proteins with the exception of BnGolS7-1 and NtGolS1-3 (Figure 1C). Motifs 4, 5, 6 and 11 were also observed in most GolS proteins. Motif 7 was detected in 30 GolS proteins and displays a specific peptide sequence –APSAA– in the C-terminus but was not found in AtGolS7, BnGolS4-2, BnGolS4-4, BnGolS7-1, BnGolS7-2 and NtGolS2-2. Motifs 8 and 12 were only detected in the N-terminus of proteins from the BnGolS6 clade, while proteins from other clades have motif 10 (Figure 1C). Motif 9 was found in the BnGolS1, BnGolS2 and BnGolS3 branches and the remaining motifs were identified in individual proteins. BnGolS1-1 and BnGolS1-4 had the most motifs, each containing 11 motifs, while BnGolS7-1 had the fewest, containing only 2 (Figure 1C). According to the InterProScan annotation, we found that motifs 1–4 are conserved and important to maintain binding activity of GolS proteins, especially the motif 2 (HxxGxxKPW) and motif 3 (DxD). 

### 2.4. Chromosome Location and Duplication Analysis

**Figure 4:**
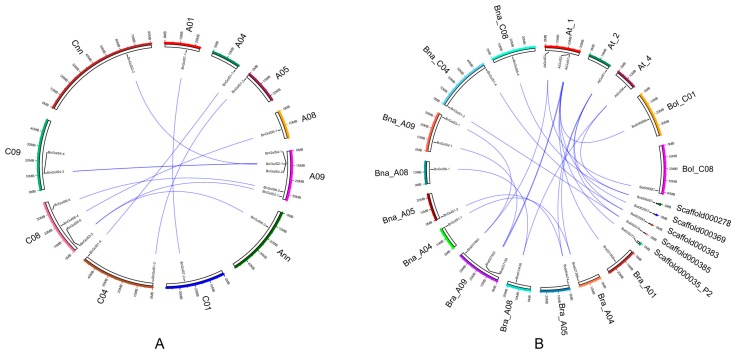
Distribution of *BnGolS* gene family members along the *B. napus* chromosomes and synteny map of *GolS* genes from *A. thaliana B. napus*, *B. rapa* and *B. oleracea.* (**A**) Chromosomal information for *BnGolS* genes was obtained from the *Brassica* database and was mapped to *B. napus* chromosomes. Syntenic relationships are indicated with connecting lines; (**B**) Genes located within the *B. napus* genome that are syntenic with genes of *A. thaliana*, *B. rapa* and *B. oleracea* are indicated by connecting lines.

In the *Brassica* database, we determined that the positions of 20 *BnGolS* genes are located on 11 chromosomes in *B. napus*. No tandem duplication events occurred in *BnGolS* gene family. No *BnGolS* genes were observed on chromosomes A02, A03, A06, A07, A10, C02, C03, C05, C06, or C07 and only one *BnGolS* gene was identified on each of the A01, A04, A05, A08 and C01 chromosomes (Figure 4A). Two *BnGolS* genes were found on chromosomes C04 and C09, four *BnGolS* genes were found on chromosome C08 and five *BnGolS* genes were found on chromosome A09. Among the four members of the *BnGolS4* subfamily, *BnGolS4-1* and *BnGolS4-3* and *BnGolS4-2* and *BnGolS4-4* were found to be closely linked on their respective chromosomes (Figure 4A), which might be the result of a tandem duplication event during evolution. Unlike the *BnGolS* genes, only four *NtGolS* genes were identified on known chromosomes, while the remaining five *NtGolS* genes were found on a scaffold which has not yet been assembled into a chromosome (Table 1). Unlike *N. tabacum*, *B. napus* experienced an additional whole genome triplication (WGT) event in its evolutional history. By comparing the synteny of *GolS* genes in *A. thaliana*, *B. napus*, *B. oleracea* and *B. rapa*, we found that some genes were preserved and some were either duplicated or lost (Figure 4B). We determined that *AtGolS1* has two homologous syntenic gene pairs in *B. oleracea*, *B. rapa* and four in *B. napus*, two of which are located on the A genome and two on the C genome. *AtGolS7* also has two homologous syntenic genes in *B. oleracea* and *B. rapa* but no syntenic homologous genes in *B. napus*. *AtGolS2* and *AtGolS3* only possesses one homologous syntenic gene each in both *B. oleracea* and *B. rapa*, that were also syntenic with homologous genes in the *B. napus* genomes A and C, respectively. We were unable to identify any syntenic homologous genes for the *AtGolS4* genein *Brassica* species (Figure 4B). 

We discovered that most of the syntenic *GolS* genes are clustered on chromosome 1 in *A. thaliana*, however, in *Brassica* most of the syntenic *GolS* genes are located on different chromosomes. *AtGolSs* have good syntenic relationships with *Brassica GolS* genes due to either genome rearrangement or gene loss after a genome triplication event. This indicates that the expansion pattern of the *GolS* gene family might be a consequence of mesopolyploidy in *Brassica* evolution [38]. We discovered 5 syntenic genes in *A. thaliana*, corresponding to 7 genes in *B. oleracea* and *B. rapa* and 8 genes in *B. napus*. These results suggest that some *GolS* members were lost after the genome triplication event in *Brassica* species and some new *GolS* members were also presented throughout its evolutionary history, which might be necessary for *Brassica* crops to cope with abiotic stresses that occur due to a change in the environmental conditions. To explore the selective pressures on these *GolS* genes, we determined the non-synonymous/synonymous mutation ratio (*K*a/*K*s) for *BnGolS* and *NtGolS* genes. Previous report showed that the patterns of codon usage bias in dicot plants are conservative [39], suggesting the *K*a/*K*s results in our study could reflect selection pressure on the evolution of three plant species. The ratios for all *BnGolS* and *NtGolS* genes were less than 1, indicating that all *BnGolS* and *NtGolS* genes were subject to purifying selection (Table 2) [40]. In *B. napus*, *BnGolS1*, *BnGolS2*, *BnGolS4* and *BnGolS7* in the A genome displayed a higher Ka/Ks ratio than their corresponding homologs in the C genome. However, the opposite was observed for *BnGolS3* and *BnGolS6*, suggesting that different evolutionary pressures were experienced on homologous genes after the whole genome triplication (WGT) in *B. napus*. The *K*a/*K*s ratio for *BnGolS1-1* and *BnGolS1-4* was significantly higher than that of other *BnGolS* family members, especially compared with *BnGolS1-2* and *BnGolS1-3* (Table 2), implying that *BnGolS1-1* and *BnGolS1-4* evolved faster than did the other *BnGolS* members and therefore, they are likely to have different biological functions. All of the *NtGolS* genes from *N. tabacum* had low *K*a/*K*s ratios, the highest of which (*NtGolS*2-6) was still lower than the lowest ratio of the *BnGolS* genes (*BnGolS*2-6) (Table 2). The *K*a/*K*s ratio of *BnGolS* was larger than that of *NtGolS*, indicating that the evolutionary pressures on *BnGolS* genes were greater than those on *NtGolS* genes. 

### 2.5. Structure Prediction and Homology Modeling

**Figure 5:**
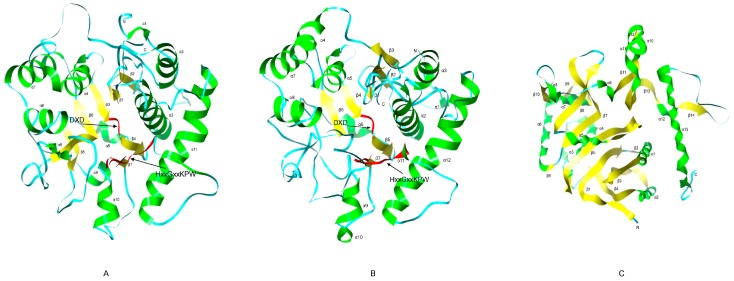
3D Structure predictions for BnGolS and NtGolS. BnGolS1-2 and NtGolS1-1 were selected as the representative GolS proteins from *B. napus* and *N. tabacum*, respectively. The models were predicted by I-TASSER and the rabbit muscle glycogenin structure (PDB ID1ll0) was used as the template for the 3D structure predication. The conserved DXD and HxxGxxPW motifs are marked on the 3D structure in red. Green represents α-helices, yellow represents β-strands and navy blue represents random coils. (**A**) Modeled3D structure of BnGolS1-2; (**B**) Modeled 3D structure of NtGolS1-1; (**C**) Template model of 1ll0B. Structural images were generated with Chimera 1.2.

To generate the 3D structures for BnGolS and NtGolS, structures for two representative GolS proteins (BnGolS1-2 and NtGolS1-1) were predicted using I-TASSER [41]. The best template used for the 3D structure predication was the rabbit muscle glycogenin [42]. The modeled structure for BnGolS1-2 has 11 α-helices and 6 β-strands (Figure 5A), while that of NtGolS1-1 has 12 α-helices and 7 β-strands (Figure 5B). The DxD motif is located between β3 and β4 in BnGolS1-2 (Figure 5A) and between β4 and β5 in NtGolS1-1(Figure 5B). Template-structure alignment resulted in a better structure match but the two GolS1 sequences had low levels of similarity with the template sequence. In the threading aligned region, the sequence identity between BnGolS1-2 or NtGolS1-1 and the 1ll0B template is 0.27 in the threading region and 0.23 across the whole template, in both cases. The coverage of threading alignment was 0.75 for BnGolS1-2 and 0.76 for NtGolS1-1. The normalized *Z*-scores of the threading alignments were both greater than 1 (1.76), meaning a good alignment was obtained for both BnGolS1-2 and NtGolS1-1 and the two predicted protein structures displayed the same overall folds. Compared with the template model, the unaligned sequences that were used to construct the models were located at the N-terminal start, C-terminal tail and a long middle sequence located on the periphery of the protein structure core.

### 2.6. Binding Pocket Prediction

**Figure 6:**
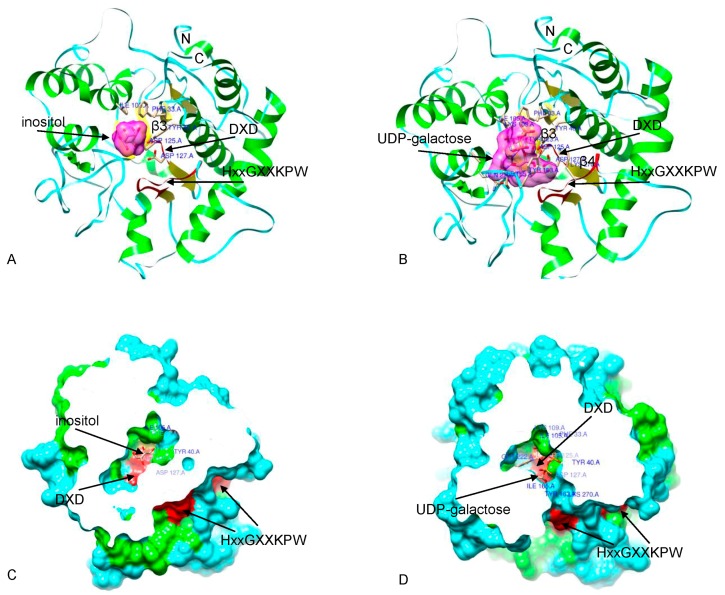
Docking of ligands onto the modeled BnGolS1-2 protein structure. (**A**) Docking results with inositol; (**B**) Docking results with UDP-galactose; (**C**,**D**) Surface representation of BnGolS1-2 showing that the ligands are buried deep in the binding pocket. In BnGolS1-2 proteins, the binding positions for UDP-galactose and inositol are close to the DxD motif. The inositol and UDP-galactose binding sites are represented in blue and the DXD and HxxGxxPW motifs are marked on the surface in red. Images were generated with Chimera 1.2.

Considering it is very difficult to predict the function of conserved motifs without a crystal structure or explicitly defined structural motifs, we applied a method that expounds the function of predicted conserved sequences by mapping conserved residues onto a sufficiently close known crystal structure [24]. Since the modeled 3D structures of the two GolS1 proteins are extremely similar (Figure 5A,B), only BnGolS1-2 was analyzed further. The important substrate-binding residues of BnGolS1-2 were predicted using the crystal structures 1zdgA and 3rmwA as templates [43]. According to this model, Asp_125_, Asp_127_, His_263_ and Cys_265_ are involved in Mn^2+^ binding, while Phe_33_, Leu_34_, Gly_36_, Asn_37_, Tyr_40_, Ile_105_, Asp_125_, Gly_126_, Asp_127_, Asn_191_, Ala_189_, Gly_193_, Ala_220_, Glu_221_, Gln_222_, His_263_, Cys_265_, Ala_266_ and Lys_270_ are involved in UDP-sugar binding. GolS proteins are known to catalyze the synthesis of galactinol from UDP-galactose and myo-inositol but no inositol-binding site was identified using I-TASSER. To support the UDP-galactose binding site and to predict the binding site for inositol, low-resolution protein structures were analyzed by the blind molecular docking method BSP-SLIM [44]. We found that UDP-galactose and inositol are both positioned near the conserved DxD motif (Figure 6A,B), which is buried deep inside the binding pocket (Figure 6C,D). Furthermore, two Asp residues play a key role in the binding of the ligands in UDP-galactose and inositol. We found that the crucial inositol-binding residues are Phe_33_, Try_40_, Ile_105_, Asp_125_ and Asp_127_ (Figure 6A), while the important UDP-galactose binding residues are Phe_33_, Try_40_, Ile_105_, Lys_109_, Try_123_, Asp_125_, Asp_127_, Try_163_, Ile_165_, Gln_222_ and Lys_270_ (Figure 6B). The Lys_270_ residue involved in UDP-galactose binding is part of the HxxGxxKPW motif. These results suggest that the conserved DxD motif plays a crucial role in the binding of micro-molecules in the galactinol catalytic pocket, whereas the HxxGxxKPW motif generally participates in macro-molecular binding. Interestingly, we found that the core area is surrounded by abundant loose coil structures, which maintain the stability of the protein and provides a stable environment for the synthesis of galactinol by preventing the hydrolysis of galactinol during the synthesis process.

### 2.7. Tissue-Specific and Hormone-Induced Expression Patterns of BnGolS and NtGolS

**Figure 7:**
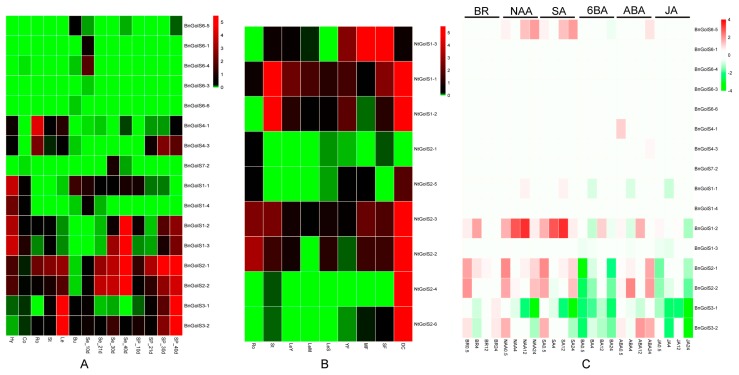
Tissue-specific and hormone-induced expression patterns of *BnGolS* and *NtGolS* genes. (**A**) Expression profiles of *BnGolS* genes in *B. napus*; (**B**) Expression profiles of *NtGolS* genes in *N. tabacum*; (**C**) Expression profiles of *BnGolS* genes in response to hormone treatments. The color bar to the right of the figures represents the log_2_ expression value and the green color represents the low or no expression in (**A**) and (**B**) while it represents the down-regulation in hormone treatment in (**C**).

To begin the investigation into the biological functions of BnGolS and NtGolS enzymes, we assessed the expression profiles of the corresponding *GolS* genes in various tissues during multiple developmental stages. In *B. napus*, the *BnGolS* families displayed divergent expression patterns. Four *BnGolS1* members were primarily expressed in hypocotyls, while *BnGolS1-1* and *BnGolS1-3* were highly expressed in seeds at 40 days after flowering (Figure 7A). *BnGolS2* members were strongly expressed in seeds and silique pericarps, especially at 40 days after flowering (Figure 7A) and high expression of *BnGolS3* members was observed in mature leaves and silique pericarps at 40 days after flowering. One *BnGolS4* member (*BnGolS4-1*) displayed abundant expression in roots. *BnGolS6* and *BnGolS7* members were not expressed in most tissues and were only weakly expressed in specific tissues during a specific development stage (Figure 7A). For example, *BnGolS6-4* was weakly expressed in seeds at 10 days after flowering and *BnGolS7-2* was weakly expressed in seeds at 30 and 40 days after flowering. In *N. tabacum*, *NtGolS2-2*, *NtGolS2-3* and all of the *NtGolS1* members displayed constitutive expression in all tissues tested (Figure 7B). *NtGolS1-1* and *NtGolS1-2* were highly expressed in stems and dry capsules, while *NtGolS1-3* was highly expressed in mature flowers and senescent flowers. *NtGolS2-2*, *NtGolS2-3*, *NtGolS2-4* and *NtGolS2-6* displayed high expression in dry capsules, while *NtGolS2-1* and *NtGolS2-5* had no detectable expression (Figure 7B). These results show that *GolS* orthologues between *B. napus* and *N. tabacum* have different tissue-specific expression patterns and that the expression profiles of the duplicated *GolS* genes in a small *GolS* gene family diverged.

To investigate the roles of the *GolS* genes in response to hormone treatments, rapeseed seedlings were subjected to brassinosteroid (BR), α-naphthaleneacetic acid (NAA), salicylic acid (SA), 6-benzyladenine (6BA), abscisic acid (ABA)and methyl jasmonate (MeJA) treatments. Six hormone inducible marker genes all showed up-regulation in response to corresponding hormone treatments (Figure S1), suggesting the physiological status of seedling plants were suitable for detecting the hormone inducible expression patterns to *GolS* genes. Although *BnGolS2s*, *BnGolS3s* and *BnGolS1-2* were strongly induced by more than one hormones, some of the remaining genes were specifically activated by a single hormone (Figure 7C). Moreover, *BnGolS1-2* was significantly up-regulated in response to NAA and SA treatments, particularly by 4 and 12 h of treatment. Two *BnGolS2* members were up-regulated in response to BR, NAA, SA and ABA treatments and were repressed in response to 6BA and JA treatments but the inducible expression was at one or two stages of hormone treatments in this study regardless of whether up-regulated or repressed. *BnGolS3-1* expression was reduced in response to NAA, SA, 6BA and JA treatments, while *BnGolS3-2* expression was reduced in response to 6BA and JA treatments (Figure 7C). Although *BnGolS6-5* expression was extremely low in all tissues examined, its expression was up-regulated in response to NAA and SA treatments. In addition, the remaining *GolS* genes were slightly induced or repressed in response to other hormone treatments but the degree of expression variation was not as dramatic as it was for the abovementioned genes (Figure 7C).

## 3. Discussion

### 3.1. Structural Characteristics of the BnGolS and NtGolS Gene Family

As representative members of the glycosyltransferase family, *GolS* genes have been lost from plant genomes over evolutionary time to varying degrees [24]. In this study, we identified 20 *BnGolS* and 9 *NtGolS* genes in *B. napus* and *N. tabacum*, respectively. We found that most of the *GolS* family members have three or few introns and most homologous genes have a similar gene structure, implied the similar function of homologous genes. In addition, most of the BnGolS and NtGolS proteins have a pI of <7, which may explain why GolS is enzymatically active in acidic conditions. GolS proteins localized to the cytoplasm, which suggests a potential osmoprotectant function for GolS in this study [24]. The conserved structural pocket in most of GolS proteins contains two conserved GT8 motifs, DxD and HxxGxxKPW, which are located very close to each other (Figure 5A,B). This suggests that these two motifs may function coordinately during the enzymatic reaction. The DxD motif is a conserved motif in diverse GT families [33] and is thought to interact with Mn^2+^ and mediate binding to NDP-sugar (Nucleotide diphosphate-sugar) donors [42,45]. The HxxGxxKPW motif was identified by *Arabidopsis* GAUT and GATL proteins, suggested that it would be part of the catalytic site [34]. The H residue in the HxxGxxKPW motif is thought to bind to Mn^2+^ and the G and K residues interact with the NDP-sugar donor [42]. The pentapeptide in the disordered loops at the C-terminus (APSAA) was found in most of the BnGolS and NtGolS proteins, with the exception of BnGolS4-2, BnGolS4-4, BnGolS7-1, BnGolS7-2 and NtGolS2-2. More than 30 amino acids in the C-terminus of these genes were missing when compared with other members. This illustrates that sequence loss has occurred in some members of the *GolS* family to varying degrees. Although no evidence was found in the literature that supports a function for the APSAA sequence in binding to UDP-galactose or myo-inositol, upon nucleotide sugar binding, some reorganized GT enzymes would adopt a new conformation within this disordered loop region to create a pocket for the acceptor molecule to bind [46,47]. We speculate that the C-terminus of the GolS proteins provides a stable environment for substrate binding based on the 3D structure and surface coverage of GolS proteins.

### 3.2. Phylogenetic and Evolutionary Relationship of GolS Proteins

GolS evolved to meet the RFOs biosynthesis requirements during abiotic stress [4,10,11,15,16], especially in higher plants [24]. In this study, GolS members in *B. napus*, *N. tabacum* and *Arabidopsis* were divided into four groups. Average expression levels of *GolS* genes in Group 2–4 from *B. napus* were significant higher than those in Group 1 (Figure 1A and Figure 7A, *p* value = 1.98 × 10^−5^, Student’s *t*-test), suggesting that Group 2–4 members might be more important than Group 1 for galactinol synthesis. BnGolS4-2/4 was more closely related to AtGolS7 than AtGolS4, while BnGolS4-1/3 was more closely related to AtGolS4 than to AtGolS7, indicating that the biological function of BnGolS4-2/4 might be diverged after WGT, which could be supported by the results of *K*a/*K*s values of BnGolS4-2/AtGolS4 and BnGolS4-4/AtGolS4 (Table 2). Generally, each *A. thaliana* gene has three syntenic copies in *B. rapa* and *B. oleracea* [48,49] and six syntenic copies in *B. napus*, since it was generated by the progenitor species of *B. rapa* and *B. oleracea* 7500–12,500 years ago [26]. However, 1.5–2 copies of each *GolS* gene were identified in *B. rapa* and *B. oleracea* and 2–6 copies were identified in *B. napus* [26,48,50] because of genome shrinkage or gene loss after WGT. In this study, 8 syntenic copies (from 20 copies) of *GolS* genes in *B. napus* and 7 syntenic copies (from 10–11 copies) of *GolS* genes were found in ancient chromosomes of *B. rapa* and *B. oleracea* ancestors (Figure 3B), suggesting that genome shrinkage or gene loss occurred in the *GolS* family of the three *Brassica* crops. Furthermore, no orthologues of AtGolS5 were found in the three *Brassica* species, indicating that AtGolS5 may have arisen via duplication of the gene encoding AtGolS6 specifically in *A. thaliana*. Monocots have fewer *GolS* copies than do Solanaceae and Brassicaceae [17,18] and Solanaceae have fewer copies than Brassicaceae. Considering the evolutionary history of plant species (Figure 3), we speculate that only one ancient copy of GolS was present in the last common ancestor of both monocots and dicots. In monocots, only one GolS copy remained, while three ancient dicot-specific GolS copies evolved from the pan-eudicot palaeohexaploidy event that occurred early in eudicot evolution. During evolution, an ancient GolS copy (corresponding to AtGolS5/6) was lost in the Solanaceae and an additional GolS copy evolved through gene duplication in Brassicaceae after the split between the Solanaceae and Brassicaceae families, resulting in 4 ancestral GolS copies in the Brassicaceae. Sixty-two percent of duplicated genes in *Arabidopsis* show differences in their expression patterns, reflecting divergence in their biological functions [51]. A similar phenomenon was observed among the duplicated members of the *GolS* family, which may have caused the divergence of *GolS* functions in response to different abiotic stress conditions.

### 3.3. Expression Profile of GolS Genes

Several studies report that galactinol primarily accumulates during seed maturation and in leaves [4,52,53]. Accumulation of *BnGolS-1* (named *BnGolS1-1* in this study) in seeds during maturation is crucial for acquiring desiccation tolerance or dry weight deposition [54]. However, in this study, BnGolS1-1sightly expressed in developing seeds, its homologous genes (*BnGolS1-2*, *BnGolS1-3*, *BnGolS2-1* and *BnGolS2-2*) strongly expressed in seed maturation to meet the acquisition. It has been reported that the important function of RFOs in phloem export and carbon storage were preserved by highly expression of *GolS* in leaves, which is crucial for plant protection against cold stress condition and oxidative damage, which is also crucial for plant protection against cold stress and oxidative damage [55]. Additionally, the reduction of *GolS* expression in leaves leads to an improvement in drought tolerance [4]. In this study, *BnGolS3* subfamily members were highly expressed in leaves, while no highly expressed *GolS* genes were observed in tobacco leaves but *NtGolS1-3* was highly expressed in mature and senescent flowers. This illustrates that *GolS* genes in rapeseed are more important to resistant to abiotic stress in leaves than *GolS* genes in tobacco. Strong expression of *GolS* genes in seeds was previously associated with desiccation tolerance in developing seeds [6,53]. In this study, *BnGolS1* members were found to be highly expressed in the cotyledon during germination, suggesting that these genes might be crucial for seed germination. The expression patterns we detected also imply that *BnGolS2* and *BnGolS3* members might be involved in silique pericarp development. Most *NtGolS* members were highly expressed in dry capsules, suggesting that they play a role in seed maturation. *BnGolS* and *NtGolS* were highly expressed in seeds, silique pericarps and dry capsules [56], which is similar to *ZmGolS* genes that are predominantly expressed in seeds [18].

It has been reported that the expression of *GolS* genes increased in response to various abiotic stresses in plants [4,17,18,57]. Expression levels of *GolS* genes were up-regulated by stimulation with plant hormones [4,58]. Among the four *BnGolS1* members, only *BnGolS1-2* shows significant up-regulation in response to NAA and SA treatments, while other *BnGolS1* members were insensitive to these treatments. By contrast, *BnGolS3-1* shows significant down-regulation in response to NAA treatment. NAA promotes plant growth and prevents leaf abscission and that SA induces the production of certain proteins. Therefore, we propose that the synthesis of galactinol or raffinose might be induced by NAA and SA. Previous studies showed that *OsGolS* and *AtGolS3* are insensitive to ABA treatments [17] and that the expression of *AtGolS1* and *AtGolS2* are weakly induced by ABA treatment [4]. Transpiration in leaves is reduced by ABA treatments through stomatal closure, which is important for the plant responses to abiotic stress and pathogen attack. Our results showed that most of the *BnGolS* genes were slightly induced by ABA treatments (Figure 7C), indicating that these genes might be associated with abiotic stress and ABA-mediated signaling. These highly expressed genes were repressed upon treatments with MeJA and 6-BA. Since JA not only has a similar effect as ABA and 6-BA but also affects the transport of carbohydrates and other organic compounds, this may suppress the biosynthesis and transport of galactinol or raffinose. The expression of *BnGolS2* members was more sensitive to hormone treatments, especially to short-term hormone induction, which suggests that plants can increase ROF biosynthesis to enhance stress tolerance by rapidly up-regulating the expression of *BnGolS2* members in response to short-term stress treatments.

In summary, we found that *GolS* genes have different expression patterns that are induced by various plant hormones and that several of these genes have important roles in plant environmental adaptation.

## 4. Materials and Methods

### 4.1. Identification of GolS Genes

Genomic, coding and protein sequences from *A. thaliana* were downloaded from the Arabidopsis Information Resource (TAIR, http://www.arabidopsis.org), those from *B. napus*, *B. rapa* and *B. oleracea* were retrieved from the *Brassica* Database (BRAD, http://brassicadb.org/brad), those from *N. tabacum*, *S. lycopersicum* and *S. tuberosum* were downloaded from the Solanaceae Database (https://solgenomics.net) and those from maize, rice and *Amborella trichopoda* were obtained from Phytozome 12.0 (www.phytozome.net).

To identify *GolS* genes in the above-mentioned species, seven GolS proteins from *Arabidopsis* were used as the query sequences in a reciprocal Basic Local Alignment Search Tool Protein (BLASTP) analysis [59,60] at a threshold E-value of 1e-5 and a minimum alignment coverage of 50%. Then, all the GolS protein sequences were analyzed with Pfam scan (http://www.ebi.ac.uk/Tools/pfa/pfamscan/) to confirm the presence of a GolS-related domain (PF01501.18, Glyco_transf_8).

### 4.2. Protein Sequence Analysis

The molecular weight (MW, kDa) and theoretical isoelectric point (pI) were calculated using the ProtParam tool in ExPASy (http://web.expasy.org/protparam/, [61]). The transmembrane transport peptides were predicted by the Tmpred tool in ExPASy (http://www.ch.embnet.org/software/TMPRED_form.html/, [62]) using default parameters and signal peptides were predicted by the SignalP 4.1 (http://www.cbs.dtu.dk/services/SignalP/, [63]) server using default parameters. The subcellular localization of each protein was predicted with the MultiLoc2.0 server (http://abi.inf.uni-tuebingen.de/Services/MultiLoc2/, [64]) using the predictive method: MultiLoc2-HighRes (Plant), 10 Localization.

### 4.3. Chromosomal Locations, Gene Structure and Protein Motif Identification Analysis 

The chromosomal locations of *BnGolS* genes and the duplicated relationship between *A. thaliana* and *Brassica* species were obtained from the *Brassica* Database and those of *NtGolS* genes were obtained from the Solanaceae Database (https://solgenomics.net/organism/Nicotiana_tabacum/genome/, [28]). The Gene Structure Display Server (GSDS 2.0: http://gsds.cbi.pku.edu.cn/, [65]) was used to determine the exon/intron structures of *BnGolS* and *NtGolS*. The Multiple Expectation Maximization for Motif Elicitation program (MEME 4.12.0, http://alternate.meme-suite.org, [37]) was used to identify the conserved motif of *GolS* proteins in *B. napus* and *N. tabacum* with the following parameter settings: the minimal and maximal motif width were set to 6 and 100 amino acids, respectively and the number of motifs was 15. Only motifs with an e-value of <1 × 10^−10^ were kept for further motif analysis (other parameters were set to default). Then the MAST 4.12.0 was used to search for the detected motifs in the protein database [66].

### 4.4. Phylogenetic and Evolution Analysis 

Multiple sequence alignment was conducted using MUSCLE (http://www.ebi.ac.uk/Tools/msa/muscle/, [67]). The ML tree was constructed using the online PhyML server (PhyML 3.0, http://www.atgc-montpellier.fr/phyml/, [68,69]) with the JTT + G substitution model. Model of rate heterogeneity: Discrete Gamma; Number of rate categories: 4; Gamma distribution parameter α: 0.43) and 100 bootstrap replicates and was used to infer evolutionary relationships among the sequences. The model used for ML and Bayesian trees were calculated using ModelGenerator v0.85 (http://mcinerneylab.com/software/modelgenerator/). There are 56 nucleotides and 96 amino acid substitution models in the Model generator. The optimal model will be selected based on the lowest values of AIC and BIC [70]. Then, CDS sequence alignments were used to calculate the synonymous mutation rate (*K*s) and non-synonymous mutation rate (*K*a) and evolutionary constraint (*K*a/*K*s) for collinear gene pairs using the *K*a*K*s_calculator 2.0 with the GY-HKY method [71].

To validate the two ML trees, the corresponding neighbor joining (NJ) and BI trees were constructed using the same alignment of GolS proteins that was prepared with MEGA7 [72] (Figures S2A and S3A) and a new alignment prepared with MrBayes 3.2.6 (Figures S2B and S3B, http://mrbayes.sourceforge.net), respectively. A bootstrap analysis was conducted with 1000 replicates for the NJ tree construction. To generate the BI tree, the JTT + G substitution model was selected and run for 5,000,000 generations, sampling every 1000 generations with default parameters. By comparing the topologies of the NJ, BI and ML trees, the ML tree with best topology was chosen for further evolutionary analyses (Table S2).

### 4.5. Three-Dimensional Structure Prediction of GolS Proteins

The three-dimensional (3D) structures were predicted with the online I-TASSER program (http://zhanglab.ccmb.med.umich.edu/I-TASSER/, [41]). To identify structurally similar templates in the Protein Data Bank (PDB), the query sequence was first subjected to multiple rounds of threading using LOMETS [73], which is a meta-threading server with nine locally installed threading programs. Rabbit muscle glycogenin (PDB ID: 1ll0) [42] was the top structural template for our queried proteins. Then, the generated 3D model was aligned to the templates using TM-align and BSLIM was used to dock ligands to the protein structure [74,75]. The constructed model was examined and visualized by Chimera 1.2 (https://www.cgl.ucsf.edu/chimera/).

### 4.6. Plant Materials and Treatment

Seeds from rapeseed cultivar “ZS11” and tobacco cultivar “K326” were germinated in plant growth chambers (a photoperiod of 16 h at 25/18 °C day/night, 60% humidity, 250 µmoles/m^2^/s, PGC Flex; Conviron, Winnipeg, MB, Canada) and then transplanted into the field in Chongqing, China. To determine the expression patterns of *GolS* family members in different tissues and organs of *B. napus*, hypocotyls (Hy) and cotyledons (Co) were harvested at 72 h after seed germination. Roots (Ro), stems (St), mature leaves (Le) and buds (Bu) from *B. napus* were collected at the initial blooming stage, while seeds (Se) and silique pericarps (SP) were collected at 10, 21, 30 and 40 days after flowering. In tobacco, roots (Ro), stems (St), young leaves (LeY), mature leaves (LeM), senescent leaves (LeS), young flowers (YF), mature flowers (MF), senescent flowers (SF) and dry capsules (DC) were sampled from tobacco plants at 75 days after transplanting (DAT).

To determine the expression patterns of *BnGolS* genes in leaves in response to hormone stresses, 4-week-old *B. napus* seedlings were grown in a plant growth chamber under the conditions introduced above [76]. For hormone treatments, the leaves of 4-week-old seedlings were treated with 10 μM BR, 50 μM NAA, 1 μM SA, 100 μM MeJA, or 50 μM ABA (GenTel, Beijing, China), prepared in ethanol, or with 50 μM 6-BA, which was dissolved in 0.1 M NaOH using 0.1% Triton X-100. Leaves from both treated and control plants were sampled at 0.5, 4, 12 and 24 h after treatments. Leaves from plants treated with 0.1% Triton X-100 in ethanol or NaOH were used as controls. For all samples used for qRT-PCR, three biological replicates were collected, each comprising the second youngest leaves of three independent plants. All samples were immediately frozen in liquid nitrogen and stored at −80 °C until RNA isolation.

### 4.7. RNA Isolation and Quantitative Real-Time PCR (qRT-PCR) Analysis

Total RNA was extracted from all samples using an RNAprep Pure Plant Kit (Tiangen, Beijing, China) and cDNA was synthesized from 1 μg of total RNA using a PrimeScript RT Master Mix Kit (TaKaRa, Dalian, China) according to the manufacturer’s instructions. Primers for the 16 *BnGolS* genes and 6 external control genes in *B. napus* used in qRT-PCR detection were obtained from the qPrimerDB database (real-time quantitative PCR Primer Database, http://biodb.swu.edu.cn/qprimerdb) [77] and primers for the nine *NtGolS* genes were designed using Geneious 10 (Biomatters, Auckland, New Zealand) (Table S3). To monitor the physiological status of plants under different hormone treatments, six hormone inducible marker genes were used as the external controls. All of the qRT-PCR reactions were implemented as described in the MIQE guidelines [78]. Two independent biological replicates, each with three technical replicates, were implemented for each sample. Relative expression levels were calculated using the 2^−ΔΔ*C*t^ method, with *BnACT7* and *NtEF-1*α as internal controls [79].

## Figures and Tables

**Table 1:** Characteristics of the twenty BnGolS and nine NtGolS proteins identified in this study.

Gene Name	Gene ID	Orthologues in *Arabidopsis*	Chromosome Location	Number of Introns	Number of Amino Acids	Molecular Weights (Mw)	Isoelectric Point (pI)	Subcellular Localization
*BnGolS1-1*	*BnaA04g26930D*	*AT2G47180* (*AtGolS1*)	chrA04: 19029593–19030985	3	342	39.26	5.23	cytoplasmic
*BnGolS1-2*	*BnaA05g00720D*	*AT2G47180* (*AtGolS1*)	chrA05: 400739–402104	3	338	38.78	6.34	cytoplasmic
*BnGolS1-3*	*BnaC04g00330D*	*AT2G47180* (*AtGolS1*)	chrC04: 214830–216198	3	338	38.88	5.95	cytoplasmic
*BnGolS1-4*	*BnaC04g51460D*	*AT2G47180* (*AtGolS1*)	chrC04: 48728752–48730072	3	342	39.15	5.52	cytoplasmic
*BnGolS2-1*	*BnaA09g15290D*	*AT1G56600* (*AtGolS2*)	chrA09: 8894797–8896969	3	330	37.86	6.76	cytoplasmic
*BnGolS2-2*	*BnaCnng63310D*	*AT1G56600* (*AtGolS2*)	chrCnn: 63178898–63180749	3	330	37.91	6.40	cytoplasmic
*BnGolS3-1*	*BnaA09g48480D*	*AT1G09350* (*AtGolS3*)	chrA09: 32476358–32477888	4	330	38.09	6.66	cytoplasmic
*BnGolS3-2*	*BnaC08g50010D*	*AT1G09350* (*AtGolS3*)	chrC08: 4447550–4449386	4	330	38.08	7.03	mitochondrial
*BnGolS4-1*	*BnaA09g14180D*	*AT1G60470* (*AtGolS4*)	chrA09: 8110757–8112355	3	334	38.34	4.69	cytoplasmic
*BnGolS4-2*	*BnaA09g14190D*	*AT1G60470* (*AtGolS4*)	chrA09: 8119504–8120800	3	297	34.69	5.10	cytoplasmic
*BnGolS4-3*	*BnaC09g14710D*	*AT1G60470* (*AtGolS4*)	chrC09: 11303590–11304914	3	334	38.37	4.70	cytoplasmic
*BnGolS4-4*	*BnaC09g14720D*	*AT1G60470* (*AtGolS4*)	chrC09: 11312323–11313549	3	297	34.68	5.11	cytoplasmic
*BnGolS6-1*	*BnaA08g14430D*	*AT4G26250* (*AtGolS6*)	chrA08: 12234029–12235330	3	337	38.77	4.89	cytoplasmic
*BnGolS6-2*	*BnaA09g41310D*	*AT4G26250* (*AtGolS6*)	chrA09: 28893974–28895377	3	336	38.43	4.87	cytoplasmic
*BnGolS6-3*	*BnaAnng12190D*	*AT4G26250* (*AtGolS6*)	chrAnn: 13192498–13193777	3	338	38.85	5.13	cytoplasmic
*BnGolS6-4*	*BnaC08g12130D*	*AT4G26250* (*AtGolS6*)	chrC08: 17362334–17362334	3	337	38.74	4.94	cytoplasmic
*BnGolS6-5*	*BnaC08g33920D*	*AT4G26250* (*AtGolS6*)	chrC08: 32207643–32209044	3	336	38.51	4.92	cytoplasmic
*BnGolS6-6*	*BnaC08g50120D*	*AT4G26250* (*AtGolS6*)	chrC08: 4493099–4494386	3	337	38.79	5.00	cytoplasmic
*BnGolS7-1*	*BnaA01g22350D*	*AT1G60450* (*AtGolS7*)	chrA01: 14694496–14694861	0	121	13.77	6.37	cytoplasmic
*BnGolS7-2*	*BnaC01g28520D*	*AT1G60450* (*AtGolS7*)	chrC01: 26460047–26467911	3	331	37.87	5.05	cytoplasmic
*NtGolS1-1*	*Nitab4.5_0000222g0170*	*AT2G47180* (*AtGolS1*)	chr19: 69267186–69268901	3	343	39.24	5.63	cytoplasmic
*NtGolS1-2*	*Nitab4.5_0001013g0090*	*AT2G47180* (*AtGolS1*)	chr22: 93811889–93813765	3	343	39.34	5.51	cytoplasmic
*NtGolS1-3*	*Nitab4.5_0003324g0150*	*AT2G47180* (*AtGolS1*)	Scaffold0003324: 299742–303780	3	223	25.26	6.52	cytoplasmic
*NtGolS2-1*	*Nitab4.5_0000136g0290*	*AT1G56600* (*AtGolS2*)	chr17: 88626458–88628277	2	293	33.50	6.95	cytoplasmic
*NtGolS2-2*	*Nitab4.5_0000178g0340*	*AT1G56600* (*AtGolS2*)	chr17: 192992786–192996672	4	360	41.40	5.94	cytoplasmic
*NtGolS2-3*	*Nitab4.5_0001617g0060*	*AT1G56600* (*AtGolS2*)	Scaffold0001617: 393036–394255	2	333	37.88	5.18	cytoplasmic
*NtGolS2-4*	*Nitab4.5_0003044g0080*	*AT1G56600* (*AtGolS2*)	Scaffold0003044: 139690–141464	2	321	36.57	6.71	cytoplasmic
*NtGolS2-5*	*Nitab4.5_0008397g0020*	*AT1G56600* (*AtGolS2*)	Scaffold0008397: 82555–87780	2	316	35.91	5.64	cytoplasmic
*NtGolS2-6*	*Nitab4.5_0011298g0020*	*AT1G56600* (*AtGolS2*)	Scaffold0011298: 15196–17559	3	342	39.15	7.07	cytoplasmic

**Table 2:** Nucleotide substitution rates for *BnGolS* and *NtGolS* genes.

*A. thaliana* ID	Gene Name	*BnGolS* or *NtGolS* ID	*K*a	*K*s	*K*a/*K*s
TWO-COPY LOCI					
*AT1G56600*	*BnGolS2-1*	*BnaA09g15290D*	0.0769267	0.517706	0.148591
*AT1G56600*	*BnGolS2-2*	*BnaCnng63310D*	0.0842008	0.492535	0.170954
*AT1G09350*	*BnGolS3-1*	*BnaA09g48480D*	0.0480083	0.361338	0.132862
*AT1G09350*	*BnGolS3-2*	*BnaC08g50010D*	0.0513324	0.392596	0.130751
*AT1G60450*	*BnGolS7-1*	*BnaA01g22350D*	0.113669	0.59331	0.191585
*AT1G60450*	*BnGolS7-2*	*BnaC01g28520D*	0.0921933	0.412872	0.223298
FOUR-COPY LOCI					
*AT2G47180*	*BnGolS1-1*	*BnaA04g26930D*	0.963356	1.12197	0.858632
*AT2G47180*	*BnGolS1-2*	*BnaA05g00720D*	0.0534271	0.489177	0.109218
*AT2G47180*	*BnGolS1-3*	*BnaC04g00330D*	0.055152	0.469188	0.117548
*AT2G47180*	*BnGolS1-4*	*BnaC04g51460D*	0.969139	1.1067	0.875705
*AT1G60470*	*BnGolS4-1*	*BnaA09g14180D*	0.0368275	0.313453	0.11749
*AT1G60470*	*BnGolS4-2*	*BnaA09g14190D*	0.131978	0.630796	0.209224
*AT1G60470*	*BnGolS4-3*	*BnaC09g14710D*	0.0350785	0.28371	0.123642
*AT1G60470*	*BnGolS4-4*	*BnaC09g14720D*	0.134025	0.607796	0.22051
SIX-COPY LOCI					
*AT4G26250*	*BnGolS6-1*	*BnaA08g14430D*	0.0960865	0.852638	0.112693
*AT4G26250*	*BnGolS6-2*	*BnaA09g41310D*	0.118529	0.706839	0.167689
*AT4G26250*	*BnGolS6-3*	*BnaAnng12190D*	0.0920022	0.793489	0.115946
*AT4G26250*	*BnGolS6-4*	*BnaC08g12130D*	0.0972559	0.840112	0.115765
*AT4G26250*	*BnGolS6-5*	*BnaC08g33920D*	0.122101	0.779304	0.156679
*AT4G26250*	*BnGolS6-6*	*BnaC08g50120D*	0.0875904	0.807485	0.108473
THREE-COPY LOCI					
*AT2G47180*	*NtGolS1-1*	*Nitab4.5_0000222g0170*	0.123879	3.51759	0.035217
*AT2G47180*	*NtGolS1-2*	*Nitab4.5_0001013g0090*	0.117014	3.7193	0.031461
*AT2G47180*	*NtGolS1-3*	*Nitab4.5_0003324g0150*	0.178767	3.64008	0.049111
SIX-COPY LOCI					
*AT1G56600*	*NtGolS2-1*	*Nitab4.5_0000136g0290*	0.158466	3.79229	0.041786
*AT1G56600*	*NtGolS2-2*	*Nitab4.5_0000178g0340*	0.18782	3.55922	0.05277
*AT1G56600*	*NtGolS2-3*	*Nitab4.5_0001617g0060*	0.154497	3.64605	0.042374
*AT1G56600*	*NtGolS2-4*	*Nitab4.5_0003044g0080*	0.249495	3.16828	0.078748
*AT1G56600*	*NtGolS2-5*	*Nitab4.5_0008397g0020*	0.173105	3.47601	0.0498
*AT1G56600*	*NtGolS2-6*	*Nitab4.5_0011298g0020*	0.258847	3.18928	0.081161

## Supplementary Materials

Supplementary materials can be found at www.mdpi.com/1422-0067/18/12/2768/s1.
