# Supplementary material for: Genome-Wide Identification, Evolutionary and Expression Analyses of the GALACTINOL SYNTHASE Gene Family in Rapeseed and Tobacco
Source: Int J Mol Sci. 2017 Dec 20;18(12):2768. doi: 10.3390/ijms18122768 (PMC5751367; doi:10.3390/ijms18122768)
Supplement: Supplementary file 1 [file ijms-18-02768-s001.pdf]

Supplementary Materials:

Genome-Wide Identification, Evolutionary and Expression Analyses of the *GALACTINOL SYNTHASE* Gene Family in Rapeseed and Tobacco

Yonghai Fan [1,2,†], Mengna Yu [1,2,†], Miao Liu [1,2], Rui Zhang [1,2], Wei Sun [1,2], Mingchao Qian [1,2], Huichun Duan [1,2], Wei Chang [1,2], Jinqi Ma [1,2], Cunmin Qu [1,2], Kai Zhang [1,*], Bo Lei [3,4,*] and Kun Lu [1,2,*]

[1] College of Agronomy and Biotechnology, Southwest University, Chongqing 400715, China; fyh1212@email.swu.edu.cn (Y.F.); yumengna1024@163.com (M.Y.); monky1117@email.swu.edu.cn (M.L.); z2247319@email.swu.edu.cn (R.Z.); reginasw@163.com (W.S.); absorbessence@163.com (M.Q.); dhcspring@163.com (H.D.); cw12345678@email.swu.edu.cn (W.C.); mjq2014@email.swu.edu.cn (J.M.); lion4302@163.com (C.Q.)

[2] Academy of Agricultural Sciences, Southwest University, Chongqing 400715, China

[3] Key Laboratory of Molecular Genetics, China National Tobacco Corporation, Guizhou Academy of Tobacco Science, Guiyang 550081, China

[4] Upland Flue-Cured Tobacco Quality and Ecology Key Laboratory of China Tobacco, Guizhou Academy of Tobacco Science, Guiyang 550081, China

* Correspondence: zhangkai2010s@163.com (K.Z.); leibo_1981@163.com (B.L.); drlukun@swu.edu.cn (K.L.); Tel./Fax: +86-23-6825-1264 (K.L.)

† These authors contributed equally to this work.

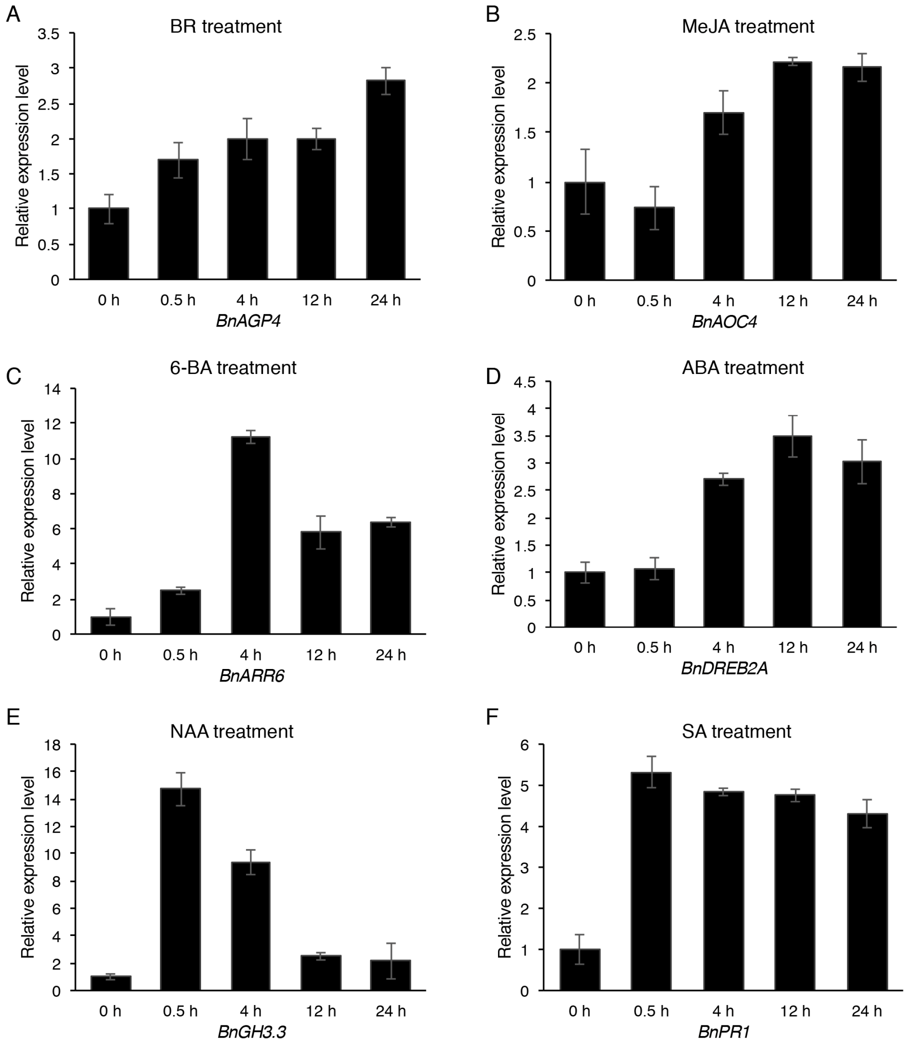

Figure S1. Expression patterns of six hormone inducible marker genes exposed to corresponding hormone treatments.

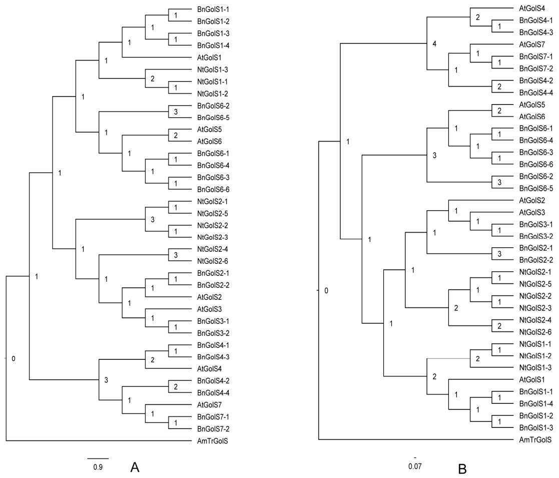

Figure S2. Phylogenetic trees of *GolS* genes in *A. thaliana*, *B. napus* and *N. tabacum*. Proteins sequences of AtGolS, BnGolS and NtGolS were aligned by using the MUSCLE program. The NJ (A) and BI (B) trees were constructed by MGEA7 with bootstrap analysis (1000 replicates) and MrBayes3.2.6. All the trees were displayed using FigTree v1.4.0.

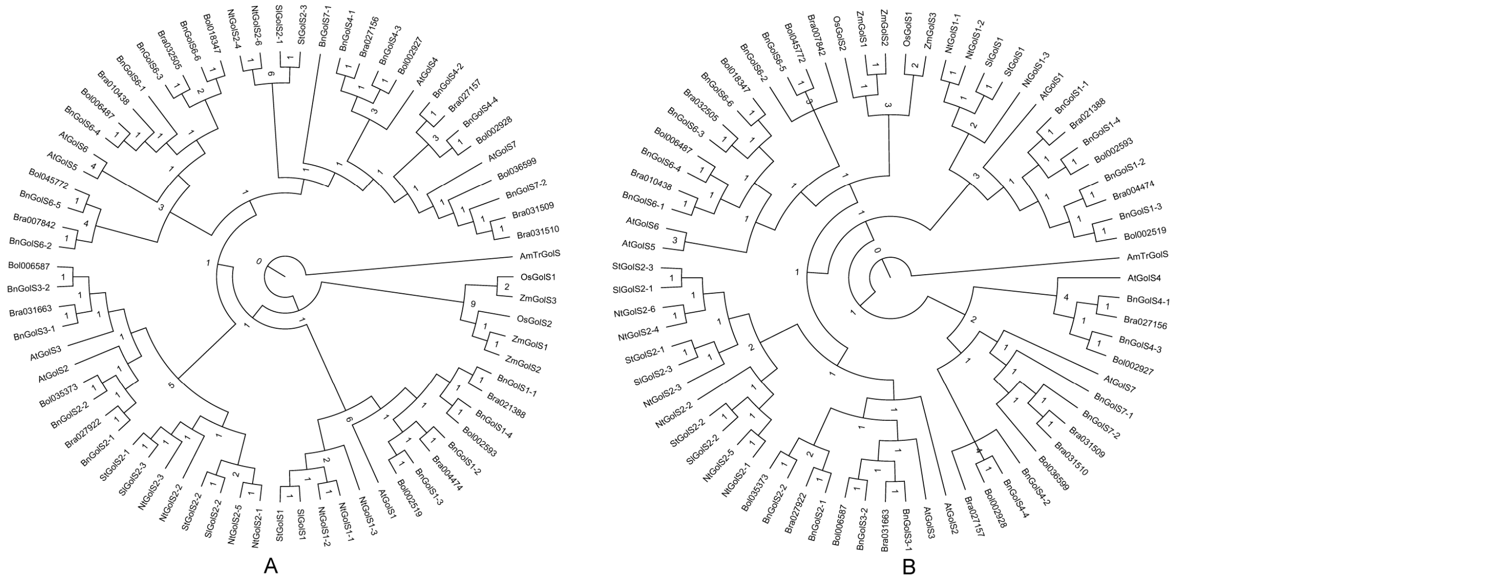

Figure S3. Phylogenetic trees of GolS proteins in plants. The NJ (A) and BI (B) trees were constructed by MGEA7 with bootstrap analysis (1000 replicates) and MrBayes3.2.6, using the GolS protiens in *A. thaliana*, *B. napus*, *B. rapa*, *B. oleracea*, *N. tabacum*, *S. lycopersicum*, *S. tuberosum*, *O. sativa*, *Z. mays* (GolS in *A. trichopoda* as an outgroup). The trees were displayed with FigTree v1.4.0. At, *A. thaliana*; Bn, *B. napus*; Bra, *B. rapa*; Bol, *B. oleracea*; Nt, *N. tabacum*; Sl, *S. lycopersicum;* St, S. *tuberosum*; Os, *O. sativa*; Zm, *Z. mays*; AmTr, *A. trichopoda*.

Table S1. Model-fit Ranking according to all measures

	AIC1	Model	Ln	AIC2	Model	Ln	BIC	Model	Ln
1	19004.56	JTT+G	-9362.28	19424.56	JTT+G	-9362.28	19536.85	JTT+G	-9362.28
2	19019.01	JTT+G+F	-9350.50	19446.56	JTT+I+G	-9370.78	19559.65	JTT+I+G	-9370.78
3	19036.22	JTT+I+G+F	-9358.11	19503.90	WAG+G	-9401.95	19623.54	JTT+G+F	-9350.50
4	19083.90	WAG+G	-9401.95	19516.22	JTT+I+G+F	-9358.11	19637.73	WAG+I+G	-9409.81
5	19101.63	WAG+I+G	-9409.81	19524.63	WAG+I+G	-9409.81	19644.56	JTT+I+G+F	-9358.11
6	19142.89	WAG+G+F	-9412.44	19611.58	Dayhoff+G	-9455.79	19723.87	Dayhoff+G	-9455.79
7	19159.58	WAG+I+G+F	-9419.79	19616.67	LG+G	-9458.33	19728.96	LG+G	-9458.33
8	19191.58	Dayhoff+G	-9455.79	19619.89	WAG+G+F	-9412.44	19736.13	DCMut+G	-9461.91
9	19196.67	LG+G	-9458.33	19623.83	DCMut+G	-9461.91	19744.02	Dayhoff+I+G	-9462.96
10	19203.83	DCMut+G	-9461.91	19630.92	Dayhoff+I+G	-9462.96	19747.42	WAG+G+F	-9412.44
11	19207.92	Dayhoff+I+G	-9462.96	19637.32	LG+I+G	-9466.16	19750.42	LG+I+G	-9466.16
12	19207.93	LG+G+F	-9444.96	19639.58	WAG+I+G+F	-9419.79	19755.05	DCMut+I+G	-9468.48
13	19209.92	Dayhoff+G+F	-9445.96	19641.96	DCMut+I+G	-9468.48	19767.92	WAG+I+G+F	-9419.79
14	19214.32	LG+I+G	-9466.16	19684.93	LG+G+F	-9444.96	19812.47	LG+G+F	-9444.96
15	19218.96	DCMut+I+G	-9468.48	19686.92	Dayhoff+G+F	-9445.96	19814.45	Dayhoff+G+F	-9445.96
16	19221.44	DCMut+G+F	-9451.72	19698.44	DCMut+G+F	-9451.72	19820.16	CPREV+G	-9503.93
17	19222.62	LG+I+G+F	-9451.31	19702.62	LG+I+G+F	-9451.31	19825.97	DCMut+G+F	-9451.72
18	19231.77	Dayhoff+I+G+F	-9455.88	19707.86	CPREV+G	-9503.93	19830.96	LG+I+G+F	-9451.31
19	19238.07	CPREV+G+F	-9460.03	19711.77	Dayhoff+I+G+F	-9455.88	19840.11	Dayhoff+I+G+F	-9455.88
20	19242.41	DCMut+I+G+F	-9461.20	19715.07	CPREV+G+F	-9460.03	19842.60	CPREV+G+F	-9460.03
21	19259.67	CPREV+I+G+F	-9469.83	19722.41	DCMut+I+G+F	-9461.20	19846.22	CPREV+I+G	-9514.06
22	19287.86	CPREV+G	-9503.93	19733.12	CPREV+I+G	-9514.06	19850.75	DCMut+I+G+F	-9461.20
23	19310.12	CPREV+I+G	-9514.06	19739.67	CPREV+I+G+F	-9469.83	19868.01	CPREV+I+G+F	-9469.83
24	19332.66	RtREV+G+F	-9507.33	19784.53	RtREV+G	-9542.26	19896.82	RtREV+G	-9542.26
25	19346.51	RtREV+I+G+F	-9513.25	19799.59	RtREV+I+G	-9547.29	19912.69	RtREV+I+G	-9547.29
26	19356.22	VT+G+F	-9519.11	19809.66	RtREV+G+F	-9507.33	19937.20	RtREV+G+F	-9507.33
27	19363.22	VT+I+G+F	-9521.61	19825.42	VT+G	-9562.71	19937.71	VT+G	-9562.71
28	19364.53	RtREV+G	-9542.26	19826.51	RtREV+I+G+F	-9513.25	19949.54	VT+I+G	-9565.72
29	19376.59	RtREV+I+G	-9547.29	19833.22	VT+G+F	-9519.11	19954.60	BLOSUM62+G	-9571.15
30	19405.42	VT+G	-9562.71	19836.44	VT+I+G	-9565.72	19954.85	RtREV+I+G+F	-9513.25
31	19413.44	VT+I+G	-9565.72	19842.30	BLOSUM62+G	-9571.15	19960.76	VT+G+F	-9519.11

32	19422.30	BLOSUM62+G	-9571.15	19843.22	VT+I+G+F	-9521.61	19968.49	BLOSUM62+I+G	-9575.19
33	19432.39	BLOSUM62+I+G	-9575.19	19855.39	BLOSUM62+I+G	-9575.19	19971.56	VT+I+G+F	-9521.61
34	19450.88	MTREV24+G+F	-9566.44	19927.88	MTREV24+G+F	-9566.44	20055.42	MTREV24+G+F	-9566.44
35	19456.05	BLOSUM62+G+F	-9569.02	19933.05	BLOSUM62+G+F	-9569.02	20060.59	BLOSUM62+G+F	-9569.02
36	19461.82	MTREV24+I+G+F	-9570.91	19941.82	MTREV24+I+G+F	-9570.91	20070.16	MTREV24+I+G+F	-9570.91
37	19466.09	BLOSUM62+I+G+F	-9573.04	19946.09	BLOSUM62+I+G+F	-9573.04	20074.42	BLOSUM62+I+G+F	-9573.04
38	19831.16	JTT+I	-9775.58	20251.16	JTT+I	-9775.58	20363.46	JTT+I	-9775.58
39	19847.61	WAG+I	-9783.80	20267.61	WAG+I	-9783.80	20379.91	WAG+I	-9783.80
40	19849.83	JTT+I+F	-9765.91	20326.83	JTT+I+F	-9765.91	20454.37	JTT+I+F	-9765.91
41	19923.41	WAG+I+F	-9802.70	20400.41	WAG+I+F	-9802.70	20527.95	WAG+I+F	-9802.70
42	19932.91	MtMam+G+F	-9807.45	20409.91	MtMam+G+F	-9807.45	20537.44	MtMam+G+F	-9807.45
43	19982.74	MtMam+I+G+F	-9831.37	20427.42	Dayhoff+I	-9863.71	20539.72	Dayhoff+I	-9863.71
44	20007.21	MtArt+G+F	-9844.60	20440.96	DCMut+I	-9870.48	20553.25	DCMut+I	-9870.48
45	20007.42	Dayhoff+I	-9863.71	20462.74	MtMam+I+G+F	-9831.37	20591.08	MtMam+I+G+F	-9831.37
46	20014.05	CPREV+I+F	-9848.02	20484.21	MtArt+G+F	-9844.60	20605.44	CPREV+I	-9896.57
47	20020.96	DCMut+I	-9870.48	20491.05	CPREV+I+F	-9848.02	20610.67	LG+I	-9899.18
48	20030.05	MtArt+I+G+F	-9855.02	20493.14	CPREV+I	-9896.57	20611.75	MtArt+G+F	-9844.60
49	20044.86	Dayhoff+I+F	-9863.43	20498.37	LG+I	-9899.18	20618.58	CPREV+I+F	-9848.02
50	20058.29	DCMut+I+F	-9870.14	20510.05	MtArt+I+G+F	-9855.02	20638.39	MtArt+I+G+F	-9855.02
51	20073.14	CPREV+I	-9896.57	20521.86	Dayhoff+I+F	-9863.43	20649.40	Dayhoff+I+F	-9863.43
52	20078.37	LG+I	-9899.18	20535.29	DCMut+I+F	-9870.14	20662.83	DCMut+I+F	-9870.14
53	20096.28	LG+I+F	-9889.14	20552.55	MTREV24+G	-9926.27	20664.84	MTREV24+G	-9926.27
54	20132.55	MTREV24+G	-9926.27	20573.28	LG+I+F	-9889.14	20700.82	LG+I+F	-9889.14
55	20173.37	VT+I+F	-9927.68	20598.97	MTREV24+I+G	-9946.98	20712.07	MTREV24+I+G	-9946.98
56	20175.97	MTREV24+I+G	-9946.98	20620.22	BLOSUM62+I	-9960.11	20732.52	BLOSUM62+I	-9960.11
57	20200.2	BLOSUM62+I	-9960.11	20649.61	RtREV+I	-9974.80	20761.90	RtREV+I	-9960.11
58	20200.22	BLOSUM62+I	-9960.11	20649.61	RtREV+I	-9974.80	20761.90	RtREV+I	-9974.80
59	20229.61	RtREV+I	-9974.80	20650.37	VT+I+F	-9927.68	20765.43	VT+I	-9976.57
60	20232.07	RtREV+I+F	-9957.03	20653.14	VT+I	-9976.57	20769.29	JTT	-9981.39
61	20233.14	VT+I	-9976.57	20657.79	JTT	-9981.39	20777.91	VT+I+F	-9927.68
62	20233.45	BLOSUM62+I+F	-9957.72	20674.64	WAG	-9989.82	20786.13	WAG	-9989.82
63	20240.79	JTT	-9981.39	20709.07	RtREV+I+F	-9957.03	20836.61	RtREV+I+F	-9957.03
64	20257.64	WAG	-9989.82	20710.45	BLOSUM62+I+F	-9957.72	20837.99	BLOSUM62+I+F	-9957.72
65	20271.21	JTT+F	-9977.60	20745.21	JTT+F	-9977.60	20871.94	JTT+F	-9977.60
66	20349.25	WAG+F	-10016.62	20823.25	WAG+F	-10016.6	20943.43	Dayhoff	-10068.4
67	20394.38	MTREV24+I+F	-10038.19	20831.94	Dayhoff	-10068.4	20949.98	WAG+F	-10016.6

68	20414.94	Dayhoff	-10068.47	20846.37	DCMut	-10075.6	20957.87	DCMut	-10075.6
69	20418.71	CPREV+F	-10051.35	20871.38	MTREV24+I+F	-10038.1	20998.82	CPREV	-10096.1
70	20429.37	DCMut	-10075.68	20887.33	CPREV	-10096.1	20998.92	MTREV24+I+F	-10038.1
71	20464.80	Dayhoff+F	-10074.40	20892.71	CPREV+F	-10051.3	21019.45	CPREV+F	-10051.1
72	20470.33	CPREV	-10096.16	20917.69	LG	-10111.3	21029.19	LG	-10111.3
73	20479.42	DCMut+F	-10081.71	20938.80	Dayhoff+F	-10074.4	21065.53	Dayhoff+F	-10074.4
74	20500.69	LG	-10111.34	20953.42	DCMut+F	-10081.7	21080.16	DCMut+F	-10081.7
75	20542.69	LG+F	-10113.34	21016.69	LG+F	-10113.3	-10113.34	VT	-10163.8
76	20560.52	VT+F	-10122.26	21022.70	VT	-10163.8	21142.28	BLOSUM62	-10167.8
77	20605.70	VT	-10163.85	21030.78	BLOSUM62	-10167.8	21142.28	BLOSUM62	-10167.8
78	20613.78	BLOSUM62	-10167.89	21030.78	BLOSUM62	-10167.8	21143.43	LG+F	-10113.3
79	20613.78	BLOSUM62	-10167.89	21034.52	VT+F	-10122.2	21152.76	MtMam+G	-10170.2
80	20620.47	MtMam+G	-10170.23	21040.47	MtMam+G	-10170.2	21161.26	VT+F	-10122.2
81	20653.37	RtREV	-10187.68	21070.37	RtREV	-10187.6	21181.86	RtREV	-10187.6
82	20658.90	BLOSUM62+F	-10171.45	-10171.4	MtArt+G	-10203.4	21219.15	MtArt+G	-10203.4
83	20685.89	RtREV+F	-10184.94	21132.90	BLOSUM62+F	-10171.4	21259.63	BLOSUM62+F	-10171.4
84	20686.86	MtArt+G	-10203.43	21159.89	RtREV+F	-10184.9	21275.82	MtMam+I+G	-10228.8
85	20739.72	MtMam+I+G	-10228.86	21162.72	MtMam+I+G	-10228.8	21286.63	RtREV+F	-10184.9
86	20788.85	MtArt+I+G	-10253.42	21211.85	MtArt+I+G	-10253.4	21324.95	MtArt+I+G	-10253.4
87	20797.62	MTREV24+F	10240.81	21271.62	MTREV24+F	-10240.8	21398.35	MTREV24+F	-10240.8
88	21246.87	MtArt+I+F	-10464.43	21709.76	MTREV24+I	-10504.8	21822.06	MTREV24+I	-10504.8
89	21289.76	MTREV24+I	-10504.88	21723.87	MtArt+I+F	-10464.4	21851.41	MtArt+I+F	-10464.4
90	21307.81	MtMam+I+F	-10494.90	21784.81	MtMam+I+F	-10494.9	21912.34	MtMam+I+F	-10494.9
91	21672.25	MTREV24	-10697.12	22089.25	MTREV24	-10697.1	22200.75	MTREV24	-10697.1
92	21685.63	MtArt+F	-10684.81	22159.63	MtArt+F	-10684.8	22286.3<br>6	MtArt+F	-10684.8
93	21745.35	MtMam+F	-10714.67	22219.35	MtMam+F	-10714.6	22346.09	MtMam+F	-10714.6
94	22302.66	MtArt+I	-11011.33	22722.66	MtArt+I	-11011.3	22834.95	MtArt+I	-11011.3
95	22344.05	MtMam+I	-11032.02	22764.05	MtMam+I	-11032.0	22876.35	MtMam+I	-11032.0
96	22754.74	MtMam	-11238.37	23171.74	MtMam	-11238.3	23283.23	MtMam	-11238.3

The optimal model selection was based on the lowest values of Akaike Information Criterion (AIC) and Bayesian Information Criterion (BIC).

Table S2. Comparison of the topologies of NJ, ML and BI trees

	item	obs	au	np	Bp	pp	kh	sh	wkh	wsh
large tree	ML	-8.7	0.741	0.725	0.725	1.000	0.848	0.917	0.848	0.946
	BI	8.7	0.261	0.275	0.275	0.152	0.152	0.344	0.152	0.271
	NJ	94.9	7e-005	4e-005	0	3e-050	5e-005	5e-005	5e-005	5e-005
small tree	ML	-0.0	0.522	0.502	0.509	0.500	0.848	0.847	0.848	0.946
	BI	0.0	0.513	0.495	0.447	0.500	0.152	0.344	0.152	0.268
	NJ	44.8	0.002	0.002	0.002	2e-020	5e-005	5e-005	5e-005	5e-005

Table S3. The primer list of 16 *BnGolS*, 9 *NtGolS* and 6 external control genes

Gene	Gene ID	Forward Primer (5′-3′)	Reverse Primer (5′-3′)
BnGolS1-1	BnaA04g26930D	CTTCGAGAAAATCTACAAGCCG	GATAAACGGCTTCAGATTCACC
BnGolS1-2	BnaA05g00720D	CACAAACCACCACAGCTAAATC	CTAAACCAACGACTCCTTTCAC
BnGolS1-3	BnaC04g00330D	TCCTCTAAAATCACGTGCTTCT	CTAAACCAACGACTCCTTTCAC
BnGolS1-4	BnaC04g51460D	GCATACGTGACATTTCTAGCTG	GGATAAGCGGATTTGACTTTCC
BnGolS2-1	BnaA09g15290D	CGACCATAAGAAGCAGTTTGTC	TCGCAAACCAGTAAAACGAATC
BnGolS2-2	BnaCnng63310D	CGACCATAAGAAGCAGTTTGTC	AATCTCAAGCCGTATCGTACAT
BnGolS3-1	BnaA09g48480D	AGGACTAAGGAAAGCTAAGAGC	CAAACTGAGTTTGGTTATCCGG
BnGolS3-2	BnaC08g50010D	CGCCTCAGTATAAGATTGGCTA	CGTTTAGAGTCTCCAAGAGGTT
BnGolS4-1	BnaA09g14180D	CGCAGTATTCCATTGGGTATTG	GGCTAGGCTCAAACAAAAACAT
BnGolS4-3	BnaC09g14710D	CGCAGTATTCCATTGGGTATTG	TCTTGTTCTGCAAAAGGTGAAG
BnGolS6-1	BnaA08g14430D	CTTCGTATTTGGGAGTTTGTGG	CGTACAAGTAACCAGAAGGAGT
BnGolS6-3	BnaAnng12190D	CGTGAAGACATAAGAATGCTGG	AGCTAAGACTGCAGTAGTGATC
BnGolS6-4	BnaC08g12130D	ACTTGTACGCGGTTAAAGATTG	AAATACGTTGGAGTGGTGATCT
BnGolS6-5	BnaC08g33920D	CCTACAACCTCGTAATGGCTAT	CCTTTGGCACAGTAATGAACAA
BnGolS6-6	BnaC08g50120D	CTTGTACGCGGTTAAAGATTGT	GACAAGATTTGGCTCAAACAGT
BnGolS7-2	BnaC01g28520D	ATGTTTGTGTTTGAGCCTAACC	GTGAACAACTTTGACTGTCTCC
NtGolS1-1	Nitab4.5_0000222g0170	CTTGATCTTGCATGGCTCCGG	CTTAGCCCCTTAGCTAACCCAAC
NtGolS1-2	Nitab4.5_0001013g0090	CATTTTCTTCAGATCACGCAACTGA	CACAATCTCTGGAGCCATGCAGGA
NtGolS1-3	Nitab4.5_0003324g0150	CATCAAACAGGGCGTATGTTACGT	CACGAGCGGATACGCAGTATGA
NtGolS2-1	Nitab4.5_0000136g0290	CCTGTTTATCCTCC-TCAGAATCAGA	TGGGCCTAACTCTTCTTCAGGC
NtGolS2-2	Nitab4.5_0000178g0340	CTTGTTGGTCTTGCAACCAAGGCTAGT	ACCCACTTGTAGTGGCTATTTTGCCT
NtGolS2-3	Nitab4.5_0001617g0060	GTTGTTGGTCTAGCAACCAAGGCT	ACCACGCCTTTCACGTAGTCACCGT
NtGolS2-4	Nitab4.5_0003044g0080	TGGCTCCTGAAGTACTTGTGAGT	ACCAACGGATACAAAGACT
NtGolS2-5	Nitab4.5_0008397g0020	CCGGTTTATCCTCCTGAGAACC	CTTTGGGCCTAACTCTTCAGCC
NtGolS2-6	Nitab4.5_0011298g0020	GGCATTGCAGTCTGAGACGT	ACCAACGGATACATAGATT
BnAGP4	BnaA07g21900D	GGTGTCGTTGTTCAATTCATGA	CAAGCCGCTAATGAATGTAACA

BnGH3.3	BnaA09g42140D	AAGTCTCCGATGATCCATACAC	GAGAAGACAAAATCATGGACCG
BnPR1	BnaC03g45470D	TTAATTTTGGCAGCCCTTGTAG	GGTTGTGAGCGTTTACATAGTC
BnAOC4	BnaA08g24350D	CCCCTCTTTTGGGTTACTCTAG	CACATGGTTCTGAAGAAACCTG
BnDREB2A	BnaC09g49920D	ACTCTTCGGAGATGTTTGATGT	GAAGTCACTACCATCTTTTGCC
BnARR6	BnaC03g51340D	AAGATCTGAGGTTTACCAAGCA	ATAAGCCCTGTTTCTCTGTCAA
